# Cybersecurity Solutions for Industrial Internet of Things–Edge Computing Integration: Challenges, Threats, and Future Directions

**DOI:** 10.3390/s25010213

**Published:** 2025-01-02

**Authors:** Tamara Zhukabayeva, Lazzat Zholshiyeva, Nurdaulet Karabayev, Shafiullah Khan, Noha Alnazzawi

**Affiliations:** 1 Department of Information Systems, L.N. Gumilyov Eurasian National University, Astana 010000, Kazakhstan; 2 College of Computing and Systems, Abdullah Al Salem University, Kuwait City 72303, Kuwait; 3 Institute of Computing, Kohat University of Science and Technology, Kohat City 24000, Pakistan; 4 Department of Computer Science and Engineering, Yanbu Industrial College, Royal Commission for Jubail and Yanbu, Yanbu Industrial City 41912, Saudi Arabia

**Keywords:** IIoT, edge computing, cyber-physical system, attack

## Abstract

This paper provides the complete details of current challenges and solutions in the cybersecurity of cyber-physical systems (CPS) within the context of the IIoT and its integration with edge computing (IIoT–edge computing). We systematically collected and analyzed the relevant literature from the past five years, applying a rigorous methodology to identify key sources. Our study highlights the prevalent IIoT layer attacks, common intrusion methods, and critical threats facing IIoT–edge computing environments. Additionally, we examine various types of cyberattacks targeting CPS, outlining their significant impact on industrial operations. A detailed taxonomy of primary security mechanisms for CPS within IIoT–edge computing is developed, followed by a comparative analysis of our approach against existing research. The findings underscore the widespread vulnerabilities across the IIoT architecture, particularly in relation to DoS, ransomware, malware, and MITM attacks. The review emphasizes the integration of advanced security technologies, including machine learning (ML), federated learning (FL), blockchain, blockchain–ML, deep learning (DL), encryption, cryptography, IT/OT convergence, and digital twins, as essential for enhancing the security and real-time data protection of CPS in IIoT–edge computing. Finally, the paper outlines potential future research directions aimed at advancing cybersecurity in this rapidly evolving domain.

## 1. Introduction

The integration of cyber-physical systems (CPS) using the IIoT brings direct connections between physical objects and corresponding digital twins into our control space, driving success and acting as a significant part of the manufacturing revolution empowered by IIoT-connected devices with networked data processing services. CPS are readily identified as a significant research area by the National Science Foundation (NSF) [1]. The IIoT is enabled by the digital technologies of industry and new system-specific devices called CPS—which form the basis for reliable device interconnection and intelligent data processing-oriented control over transducers in the IoT. The integration of CPS with the IIoT is critical for future developments in industrial automation, data abstraction from distributed devices, and security [2]. CPS merge physical processes with computational resources in such a way that the interaction between digital and physical is seamless [3,4].

The IIoT integrated the predicated and untrained physical devices with smart digital solutions using big data, which is making industrial operations more efficient. In a properly implemented industrial IoT system, the existence of networked sensors to communicate data allows devices and machines on the factory floor to run synchronously with cloud-based applications. The seamless data exchange between sensors and actuators as well as the integration of connected devices guarantee an optimal production process through IoT (IIoT) networks [5]. It uses smart technologies to allow machines to collect, detect processes, and deliver real-time events or a store-and-process architecture of carrying out the same functions. Therefore, this enhances the operation and workflow of various industries through an increase in reliability which further provides them with a competitive advantage [6].

Security is a crucial element of CPS and IIoT integration, prompting the creation of edge computing-based authentication mechanisms to safeguard against unwanted access. CPS in the IIoT have considerable security issues, as internet-connected devices are susceptible to several threats, including device tampering, DDoS assaults, malware, hacking, and other cyberattacks. These assaults can impede essential industrial operations, resulting in production halts. This requires the adoption of protective measures, such as machine learning models for anomaly detection, attack mitigation, and risk management [7]. To resolve these challenges and guarantee efficient operations, edge computing technology has been progressively evolving, processing data at the network’s periphery, thereby diminishing latency and alleviating cloud burden.

Edge computing enhances IIoT by redistributing data processing to the network’s perimeter, reducing the load on cloud resources and improving cybersecurity. This enables real-time data processing and danger detection, which is especially vital in resource-constrained environments. Edge computing reduces latency, conserves bandwidth, and accelerates responses to cyber threats, making it highly beneficial in industrial settings. Local data processing alleviates bottlenecks in wireless networks and improves the reliability of IIoT systems [8]. Artificial intelligence technologies, including deep learning (DL) and machine learning (ML), are critical elements of the IIoT (IIoT) and enhance efficiency by overseeing and regulating industrial machinery [9]. Artificial intelligence and deep learning at the edge facilitate collaborative model training among devices while ensuring data privacy. This is accomplished through technologies like federated edge learning, which enhances computing efficiency and minimizes latency [10]. Edge computing integrates with IoT, 5G, and AI technologies, enhancing sophisticated applications such as driverless vehicles and augmented reality. The principal advantages comprise diminished latency, improved bandwidth utilization, heightened data privacy and security, and energy efficiency [11,12]. Notwithstanding their benefits, the extensive deployment of edge devices renders them progressively susceptible to cyber threats, hence mandating an emphasis on cybersecurity [13].

Edge computing and cyber-physical systems significantly augment the operational capabilities of the IIoT; however, they concurrently present unprecedented challenges pertaining to security and resource management [14]. While advantages such as interoperability, enhanced security measures, and the integration of artificial intelligence are notable, substantial obstacles remain, particularly concerning the deployment of 5G technology and edge virtualization [15]. Cybersecurity emerges as a critical issue within the IIoT framework, resulting in challenges related to device authorization, authentication, and the preservation of data integrity. To ensure the protection of data transmission throughout the network, lightweight block ciphers and supplementary security protocols are implemented. The hardness for keeping system integrity forces strong security tactics like cryptographic methods and intrusion detection systems in order to stand against cyber attacks [16]. Also, standardization and compatibility must be universal in order to easily blend IIoT elements. Tackling these challenges is inherently related to setting up and enforcing standards, such as cybersecurity laws that aim at protecting machine-to-machine (M2M) interactions [17,18,19]. The requirement for real-time communication technologies is critical for the IIoT; however, the lack of well-defined protocols presents challenges to both security and reliability [20].

Contemporary approaches to enhancing the security of CPS within the IIoT context through the integration of edge computing encompass a convergence of blockchain technology, machine learning, and advanced authentication methodologies. These methodologies are carefully designed to augment the security of informational exchanges. The adoption of blockchain technology significantly enhances security due to its inherent properties of traceability, stability, and resilience, rendering it particularly suitable for IIoT initiatives [21,22,23]. This amalgamation effectively addresses several pivotal challenges, such as cybersecurity weaknesses, data privacy issues, and the identification of anomalies. The Ethereum blockchain inside EdgeBot secures the proof of data ownership to facilitate secure movement of information between external stakeholders and edge devices while preserving transaction verifiability for incentive mechanisms [24]. Recent studies have shown that blockchain technology is essential for cooperative threat detection in IIoT ecosystems by using digital twins, integrating with the anomaly detection process on a system level according to actual conditions of transactions involving IIoT and also applying machine learning algorithms when viewing anomalous patterns [25,26]. AI algorithms are essential for the security of critical infrastructure [27] and detection and mitigation methods against cyber attacks, such as worm propagation mechanisms using AI techniques [28]. However, despite the considerable appeal of these methods, there are ongoing costs associated with adaptation to new threats and constraints in knowledge from subtle effects that interfere with impact measurements.

Informed by the context, we propose the subsequent research questions:

– RQ 1. What constitutes IIoT networks and which fundamental technologies facilitate their optimal operation?
– RQ 2. In what ways does edge computing enhance the IIoT and what cybersecurity benefits are linked to its deployment?
– RQ 3. Regarding CPS inside the IIoT, what are their effects on attacks?
– RQ 4. How will edge computing secure CPS with IIoT strategies?

This paper aims to shed light on the top challenges in this space, propose novel solutions for these endeavors, and discuss the role of edge computing from a security perspective inside IIoT environments first, the latter of which is a key risk factor for industrial operations.

The contributions of this paper are as follows:

– We developed a process for detecting new and relevant articles on the topic of interest.
– We identified common IIoT layer attacks and penetration approaches.
– We found common attacks and threats in IIoT edge computing.
– We reviewed the cyberattack types over CPS and their impact on industry.
– We broke down the real security techniques CPS in IIoT–edge computing and adopted them into a taxonomy.
– A comparison study between our methodology and existing techniques in this area.

**Figure 1:**
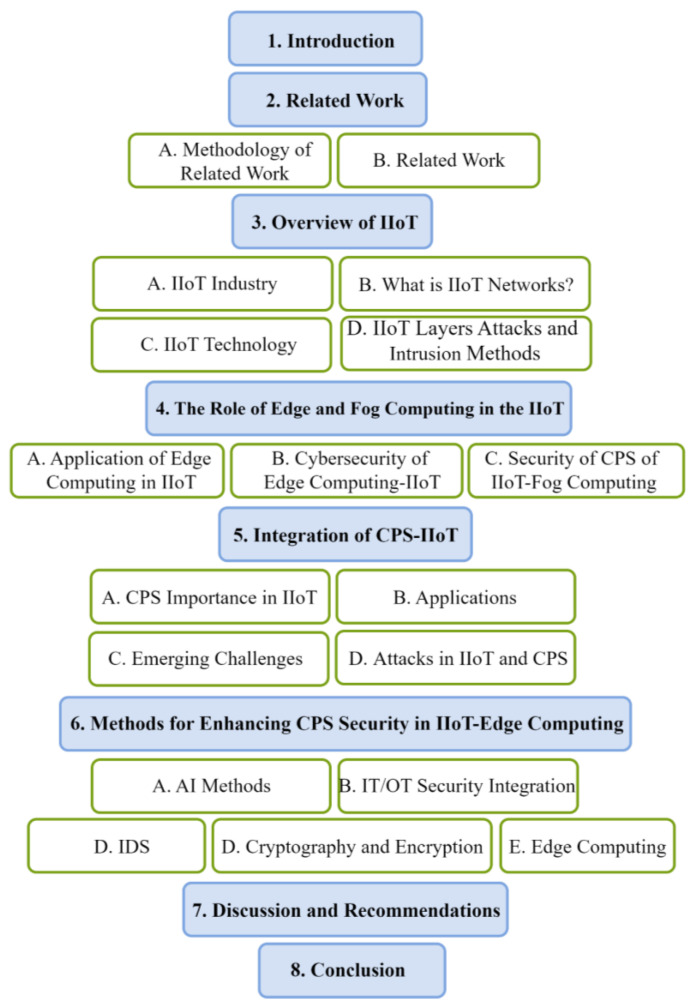
Paper structure.

Paper structure: The document is organized as follows; Section 1 starts by presenting the objectives, questions, and contributions of this study. Section 2 delineates the methodology of pertinent research. Section 3 offers a comprehensive introduction of the IIoT, encompassing networks, technologies, vulnerabilities at IIoT tiers, and techniques of entry. Section 4 analyzes the function of edge computing inside IIoT, the amalgamation of CPS with IIoT, and their security considerations. Section 5 addresses strategies for augmenting the security of CPS in IIoT–edge computing. Section 6 examines strategies to enhance the security of cyber-physical systems in the IIoT through the utilization of edge computing. Ultimately, Section 7 delineates the conclusion (Figure 1).

## 2. Related Work

This section presents key research related to the theme. Graphs show the increase in the number of publications for each set of keywords, emphasizing the significance and growing interest in the research topic in recent years.

### 2.1. Methodology of Related Work

Algorithm for study selection: A systematic literature evaluation was performed, utilizing predefined keywords and databases. The criteria that were used to search and select articles are outlined. The authors have referred to the papers published in top scientific journals and carried out extensive research on specialized scientific databases like Scopus and Google Scholar. The focus was drawn towards the last five years of publications, which represent the recent advancements in the discipline. Articles with a considerable citation index or that were published in high impact factor journals were also included. After the initial search, relevant articles were identified based on how well they address the aforementioned research questions.

The screening criteria for search queries were keywords. Industrial Internet of Things, IIoT, edge computing, cyber-physical systems, and attack were the topic words, and were combined with an attack range within 2020–2024.

Set 1: Initially, 1133/110 articles were selected from the Scopus and Google Scholar databases for the years 2020 to 2024. After filtering for the fields of engineering and computer science, and excluding all publication formats except for journal articles while also omitting 3/1 non-English items, 402/53 articles were kept.

Set 2: In total, 110/53 articles were extracted from Scopus/Google Scholar for the span 2020–2024. Upon filtering by the domains of IIoT engineering and IIoT computer science, and restricting the selection to journal papers, 61/47 articles remained.

Duplicates were found and removed, following which all included publications were examined based on their abstracts and outcomes. Upon completion of all algorithmic processes, 235 articles were identified for subsequent research.

**Figure 2:**
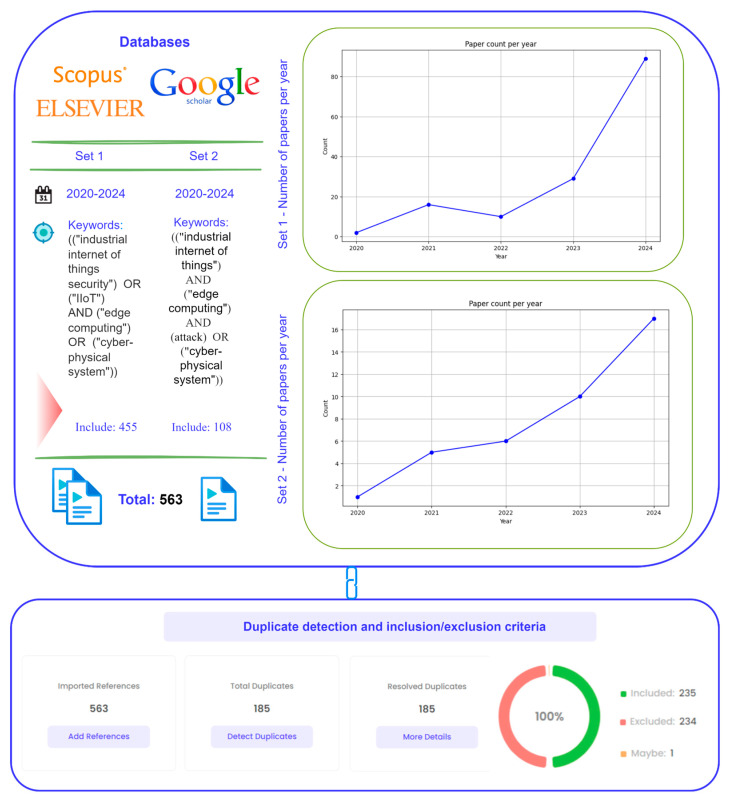
Procedure for selecting related work.

Figure 2 illustrates that, in accordance with the previously referenced data, a meticulous curation of pertinent publications was performed, with a focus on domains such as IIoT security, edge computing, and cyber-physical systems. Redundant entries were detected and systematically removed, resulting in a conclusive assembly of 235 distinct and pertinent articles for subsequent examination. Following a thorough review of the full text of all included articles, 185 were retained. The graphs highlight the rise in the number of publications for each set of keywords, reflecting the significance and growing interest in the topics studied in recent years.

### 2.2. Related Work

The lack of established protocols and the range of technologies contribute to the incompatibility of devices in the industrial IIoT, which has a significant impact on real-time data gathering and machine-to-machine communication in Industry 4.0. Resolving these challenges is essential for enhancing productivity and minimizing expenses. Various degrees of compatibility, along with advanced technologies like blockchain and 5G, can enhance data exchange and device integration in the IIoT [29]. In recent years, the IIoT has become an essential element of intelligent systems; yet, apprehensions over privacy and data security continue to arise due to the management of significant volumes of sensitive information. Conventional cloud computing is inadequate in managing latency and bandwidth, rendering edge computing a more efficient alternative [30]. The IIoT encompasses many devices and sensors that communicate data inside a sophisticated network, necessitating the use of contemporary edge computing technologies. Multiple facets of edge computing are examined, encompassing security, latency, resource utilization, and energy efficiency [31]. 

An examination of IIoT testbeds, categorized by communication protocols including TSN, IEEE 802.15.4, IEEE 802.11, and 5G, underscores their significance for evaluating innovations in authentic industrial settings. Our focus is directed on communication protocols and resource management techniques, including quality of service (QoS) prioritization and security [32]. The IIoT enhances industrial operations while simultaneously presenting substantial security dangers, necessitating dependable intrusion detection systems (IDS). Proposals have been made to utilize machine learning to improve IIoT security [33,34]. 

Future research opportunities involve enhancing the scalability and efficiency of IDS models to manage the varied and intricate data characteristic of IIoT networks [35]. Although ML-based IDS models have advanced considerably, they encounter obstacles like the requirement for extensive labeled datasets and substantial computational resources. Conversely, non-ML-based models such as IoT-PRID utilize economical network traffic and offer a lightweight alternative; however, they may not encompass all attack types [36].

The integration of AI into edge computing, through a three-tier architecture and federated learning, enhances the deployment of AI models and optimizes resource use [37]. The use of AI in Multi-access Edge Computing (MEC) streamlines model deployment, reduces training time, and saves energy [38]. However, complex AI operations need robust servers, which can increase energy consumption within the IIoT ecosystem [39]. Intelligent edge computing is proposed as a solution, providing energy-efficient AI processing for applications in the IIoT.

The rise of the IIoT has heightened the risk of cyberattacks, making it essential to implement robust defense strategies. While traditional ML techniques may struggle to address emerging threats, DL and CNN excel at identifying malware by interpreting data in a visual format. There is a system using edge computing that achieves a classification accuracy of 98.93%, confirming its effectiveness and the need for further research to combat emerging threats [40]. Intelligent edge computing also contributes to energy efficiency and reduces consumption compared to traditional methods. To enhance protection against attacks, deep learning and CNNs are used for malware analysis, and new methods such as FedGame provide effective protection and privacy [41].

IIoT necessitates data safeguarding against vulnerabilities via blockchain technology and artificial intelligence. When integrated with lightweight intrusion detection systems and advanced cryptographic algorithms, these technologies guarantee excellent precision and efficacy in IIoT systems [42]. A blockchain-based machine learning framework (BML-ES) is presented for efficient data processing and real-time transmission in the Industrial Internet of Things (IIoT) at the network edge, utilizing smart contracts and the SM2 cryptosystem to improve security and model correctness [43].

To effectively safeguard IIoT systems against growing vulnerabilities, a multi-layered architecture is crucial, integrating physical, network, and application levels [44]. This strategy emphasizes the significance of employing cryptography, intrusion detection systems, and blockchain technologies to bolster security [45]. Although these technologies greatly enhance IIoT security, there is still a pressing need for universal security solutions that can adapt to the diverse and constantly evolving landscape of IIoT systems. The rise of numerous IIoT devices amplifies threats, such as botnets, which provide attackers with tools to launch assaults. The variety and complexity of botnet attacks require efficient and timely detection. While machine learning and deep learning excel at identifying these threats, centralized models and the lack of up-to-date data present challenges. Edge computing (MEC) and federated learning (FL) represent promising solutions. Experiments have validated the exceptional accuracy and efficiency of FedGame [46]. Federated edge learning (FEL) in the Industrial Internet of Things (IIoT) encounters problems including high communication expenses and data protection issues. The integration of edge computing with software-defined networks (SDN) enhances computing services while decreasing network latency and implementation expenses [47]. A hybrid deep learning method for safeguarding IIoT infrastructure against various and intricate botnet attacks has achieved a detection accuracy of 99.94% and a response time of 0.066 ms, validating its efficacy and rapidity [48]. The AttackNet model, utilizing an adaptive CNN-GRU architecture, demonstrates exceptional proficiency in identifying botnet attacks, with a testing accuracy of 99.75% and substantially surpassing current methodologies [49]. To provide effective anomaly monitoring and detection in CPS, it is essential to use machine learning models on IIoT devices. In 6G, the digital twin (DT) generates a virtual representation of the network topology for real-time administration. In intelligent manufacturing, the IIoT utilizes mobile edge computing (MEC) to assign tasks. Many cyber-physical systems rely on outdated infrastructures that were not designed to tackle modern cyber threats. As a result, these systems often have weak security measures, making them vulnerable to attacks [50].

Table 1 presents a summary of the existing research landscape on IIoT, highlighting its advantages such as increased operational efficiency and greater cybersecurity via the incorporation of IDS into CPS with high precision. However, obstacles remain, such as the intricacies of technological integration, elevated redundancy expenses, and inadequate long-term data. ML algorithms face difficulties in threat detection due to data imbalance, and further advancements in blockchain and edge computing are essential for establishing a reliable infrastructure. The main goals continue to be reducing latency and boosting IIoT efficiency, while privacy, encryption, and scalability need thorough solutions.

## 3. Overview of Industrial Internet of Things

The IIoT is a network that links equipment and devices involved in industrial production via the internet. This connectivity enables data sharing and communication, enhancing the efficiency and quality of processes. There are two primary types of sensors utilized: edge sensors, which gather data near the machines, and connected sensors, which relay information to other devices. This setup enables quick decision-making and real-time optimization of processes. The IIoT allows for swift responses to issues, thereby minimizing downtime and boosting production. In an automotive manufacturing facility, sensors on the assembly line can identify defects and halt production to rectify the issue, so preventing the delivery of flawed products. The adoption of IIoT enhances product quality, lowers expenses, and elevates consumer satisfaction, rendering production more intelligent and efficient [62].

### 3.1. IIoT Industry

The IIoT is being utilized across various sectors, including manufacturing, transportation and logistics, energy, healthcare, smart cities, agriculture, and construction. It is set to transform these industries in the future by harnessing advancements in communication, data analytics, and automation. This transformation is driven by the integration of 5G, AI, and IoT technologies, which together enhance operational efficiency, security, and sustainability in many fields. The IIoT is crucial for building smart cities by optimizing resource management, boosting public safety, and improving transportation systems. In urban environments, implementing IIoT means utilizing autonomous devices and networks to develop intelligent grids and infrastructure, which are key to sustainable urban growth [63].

In the transportation sector, the IIoT facilitates intelligent logistics through enhanced tracking and fleet management systems. These systems enhance supply chain efficiency by delivering real-time information regarding the location, condition, and performance of vehicles [64]. Blockchain-enabled safe data exchange models guarantee the integrity and security of data transmitted inside transportation networks, mitigating the risk of data breaches and enhancing confidence among stakeholders [65].

The applications of the IIoT in the energy industry encompass smart grids and energy management systems that enhance the optimization of energy distribution and consumption. These systems depend on real-time data to enhance efficiency and decrease operational expenses. Federated learning in IIoT networks safeguards sensitive data while delivering intelligent energy management solutions, effectively addressing scalability and data privacy concerns [66].

The IIoT is integral to smart production, facilitating automation, resource efficiency, and predictive maintenance. The integration of IoT with production processes facilitates real-time monitoring and control, resulting in enhanced productivity and diminished downtime [67,68].

The construction sector gains advantages from the IIoT via enhanced project management, equipment tracking, and safety oversight. IoT devices can facilitate the monitoring of construction sites for adherence to regulations and operational efficiency, akin to other industrial applications [69].

The healthcare sector gains from IIoT through enhanced patient monitoring and management systems, which improve care quality and operational efficiency. The IIoT facilitates the incorporation of artificial intelligence technology, including machine learning, to analyze patient data and forecast health outcomes, hence enhancing proactive health management [70].

In agriculture, IIoT applications encompass precision farming, utilizing sensor and drone data to assess plant health, soil conditions, and weather patterns, hence enhancing yields and resource efficiency. The implementation of IIoT in agriculture mitigates waste and enhances the sustainability of farming methods via data analysis [71].

In mining, the IIoT enhances safety, boosts productivity, and promotes environmental sustainability by automating processes and offering a comprehensive monitoring system [72].

Although IIoT presents considerable advantages in these sectors, challenges like data security and integration complexity continue to pose significant hurdles. The large volume of data produced by interconnected devices necessitates strong security protocols to safeguard against unauthorized access and data breaches.

**Figure 3:**
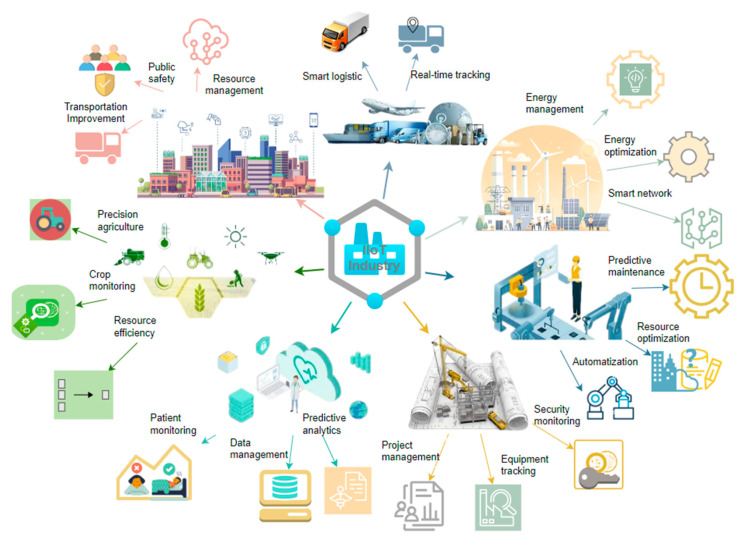
Integration of physical and digital technologies in IIoT.

The interconnected features of IIoT systems and their significant influence on various industries are depicted in Figure 3. They highlight how the IIoT merges physical processes with digital technology to improve efficiency, monitoring, and decision-making.

#### 3.1.1. Implementation Examples

A cost-effective wireless sensor network using ZigBee has been set up to monitor processes in a factory focused on solar shade production. This technology is associated with ERP systems, optimizing production processes and cost assessments [73]. The versatile platform facilitates the incorporation of sensors and data processing functionalities, hence improving the production processes. Drones integrated with IoT technology provide efficient data acquisition concerning vegetation indices, soil moisture, and crop health, essential for precision agriculture. Numerous nations have commenced the incorporation of IIoT into their industrial frameworks [74]. The German Industry 4.0 program and China’s Made in China 2025 are centered on the integration of digital technology [75]. Consequently, the incorporation of IIoT is revolutionizing industries by improving process control and automation. The Industrial Internet of Things (IIoT) possesses significant promise for resolving difficulties and enhancing the efficiency of industrial processes.

#### 3.1.2. What Are IIoT Networks?

IIoT networks enhance product quality, optimize machine functionality, reduce costs, and increase operational efficiency. To attain maximal efficiency, an IIoT network must execute two essential functions: creating connections between devices and a centralized system, and facilitating the storage, management, analysis, and effective utilization of the collected and sent data. These networks comprise numerous interconnected industrial devices, sensors, and systems that methodically gather, disseminate, and analyze data to optimize industrial processes and support informed decision-making.

#### 3.1.3. Structure and Components of IIoT Networks

The framework of IIoT networks is structured in several layers: the perception layer, which includes sensors for gathering data; the network layer, responsible for connectivity and data transmission; the processing layer, which analyzes the data and generates inferences; and the application layer, where actionable insights are provided to end users. In this setup, sensors in the perception layer gather data, the network layer maintains connectivity, the processing layer takes care of data analysis, and the application layer communicates the results to users. Moreover, IIoT platforms foster collaboration between humans and machines, which enhances operational efficiency and productivity in manufacturing. 

These outcomes emphasize the vital role that IIoT plays in transforming data collection, connectivity, decision-making, and human–machine interaction within the manufacturing industry [76]. By integrating machine-to-machine (M2M) communication technologies with cyber-physical systems, IIoT facilitates the real-time monitoring and automated control of devices [77].

**Figure 4:**
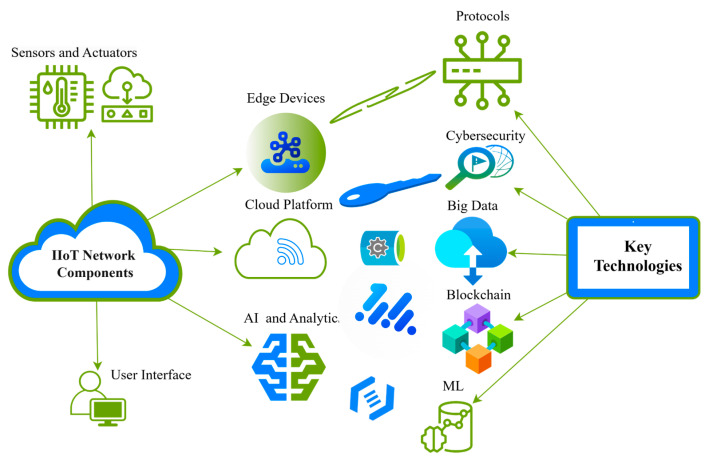
Interaction of key IIoT components and technologies.

Figure 4 illustrates the interplay of essential components and technologies in IIoT employed for the automation of industrial processes. In an IIoT network, sensors gather data from the physical environment, while actuators execute actions based on these data. Edge devices locally process data, minimizing latency and network congestion, but the cloud platform offers extensive data storage and analytical capabilities. Technologies such as artificial intelligence (AI), machine learning (ML), and blockchain significantly enhance predictive accuracy, bolster data security, and improve process management efficiency. Cybersecurity safeguards the system against attacks, whereas communication protocols provide dependable data transmission between devices, upholding elevated standards of reliability and speed in industrial settings.

#### 3.1.4. Security and Data Management

Security is a crucial component of the IIoT, particularly regarding its connection with cloud computing. Significant vulnerabilities encompass side-channel attacks. Risk mitigation strategies, including HyperSafe, intrusion detection systems (IDS), and encryption techniques, are essential for safeguarding the security and integrity of IIoT infrastructure. Information protection is an essential component of IIoT networks, primarily because of the pervasive hazards posed by hackers. Advanced deep learning architectures, such as convolutional neural networks (CNN) and gated recurrent units (GRU), are employed to create anomaly detection systems, therefore improving the security of these networks [78]. Efficient data management is crucial for managing substantial data volumes, guaranteeing the stability and scalability of IIoT networks [79]. 

### 3.2. IIoT Technology

The incorporation of new technologies, including 5G/6G, IIoT sensors, cloud and edge computing, artificial intelligence, machine learning, and cybersecurity protocols for cyber-physical systems, is essential for improving the functionality and security of IIoT systems. These technologies jointly enhance the optimization, efficiency, and security of industrial operations.

#### 3.2.1. Fifth-Generation and 6G Technologies

Given that IIoT networks necessitate the transmission and reception of substantial data quantities produced by machines and devices, this functionality was formerly restricted to Wi-Fi connectivity. The emergence of 5G technology and other advancements in cellular networks has transformed this paradigm, enhancing the bandwidth required for managing extensive datasets while concurrently decreasing latency and energy usage. Sixth-generation technologies, including cloud-based XR and digital twins, are poised to transform IIoT by delivering the requisite high-speed, low-latency connectivity for real-time applications. 

#### 3.2.2. IIoT Sensors

The incorporation of IoT gateway components, like cameras and sensors, into conventional machinery enhances monitoring and augments the intelligence of industrial operations. Embedded sensors provide the real-time surveillance of environmental parameters and operational metrics, enhancing automation and the development of digital twins, hence augmenting process predictability [80,81,82].

#### 3.2.3. Cloud and Edge Computing

Cloud infrastructure and edge computing are integral to augmenting IIoT networks by supplying computational resources and storage, while facilitating local data processing. This amalgamation facilitates the effective processing of extensive datasets, minimizing latency and permitting real-time data management. The amalgamation of these technologies facilitates a dynamic and efficient data management system, enabling the local processing of data at the edge before transmission to AI-driven centralized systems for comprehensive analysis. Edge computing markedly enhances processing performance in IoT systems by minimizing latency. Experiments indicate that edge computing can decrease average latency from 4.03 milliseconds to 3.03 milliseconds, demonstrating its efficacy in streamlining data processing and analysis [83]. Furthermore, edge computing frameworks such as federated learning-oriented systems mitigate challenges related to device connectivity and power consumption by facilitating the deployment of AI models at the edge, therefore decreasing both latency and energy expenditures [84]. Local data processing decreases network congestion and improves privacy by enabling sensitive data to be processed at its origin without the necessity of transmission to the cloud [85]. Cloud architecture facilitates the aggregation and examination of extensive data streams from IIoT networks, hence enabling resource-intensive machine learning activities. The amalgamation of cloud and edge resources facilitates dynamic equilibrium between local processing and centralized analysis, enhancing resource utilization and augmenting system scalability [86]. Although edge computing offers considerable benefits in minimizing latency and improving privacy, it may result in diminished model accuracy due to the limited training data accessible at the edge [87]. A balanced strategy that utilizes both edge and cloud capabilities is essential for optimizing IIoT network performance and facilitating thorough data analysis.

#### 3.2.4. Artificial Intelligence and Machine Learning

The incorporation of AI and ML into IIoT data processing markedly improves efficiency, competitiveness, and customer happiness. Contemporary algorithms enable firms to handle diverse data kinds, including unstructured information, thereby enhancing analytics and decision-making. Machine learning methodologies are essential for analyzing substantial quantities of Internet of Things data, identifying anomalies and dangers in real-time and thereby improving system security and dependability. The incorporation of AI and machine learning streamlines company operations, decreases expenses, and enhances scalability in dynamic sectors. AI models in IIoT systems enhance industrial processes via defect prediction, process optimization, and predictive maintenance. These models enhance operational efficiency and elevate product quality control, offering extensive insights into AI-driven IIoT systems [88]. Artificial intelligence techniques, including rule-based reasoning and reinforcement learning, are employed to identify and mitigate attacks on IIoT devices, thereby improving security and resilience. Despite substantial advancements in IIoT data processing and business processes facilitated by AI and machine learning, obstacles persist, including data privacy, algorithmic bias, and the necessity for regular model upgrades [89]. 

### 3.3. IIoT Layers Attacks and Intrusion Methods

The IIoT is vulnerable to multiple types of attacks that can profoundly affect industrial processes. Attacks may manifest at several tiers of the IIoT architecture, encompassing the perception, network, and application layers, each possessing distinct vulnerabilities and repercussions. Methods for prevention and mitigation are also summarized in Table 2.

#### 3.3.1. Application Layer Attacks

The application layer of IIoT is especially susceptible to diverse cyberattacks owing to its intricate and linked characteristics. At this tier, assailants can employ advanced techniques, such as bogus data injection, to evade detection while altering sensor data. These assaults aim at the software and apps operating on IIoT devices, potentially resulting in illegal control and data manipulation and hence compromising the entire functionality of industrial systems. Such attacks can impede operations, undermine data integrity, and inflict considerable financial and reputational harm. IIoT systems are particularly susceptible to ransomware attacks because of their dependence on networked equipment and protocols. Cascading assaults transpire when the interplay among numerous devices and services, frequently enabled by platforms such as IFTTT, generates vulnerabilities. The escalating utilization of IIoT devices, particularly with the emergence of 6G technologies, heightens the potential of data breaches and infringements of privacy. The interrelated characteristics of these devices render them vulnerable to illicit access and data exfiltration. Eavesdropping and spoofing attacks entail the interception and modification of communications between devices [90]. Eavesdropping enables attackers to obtain unauthorized access to confidential information, whereas spoofing entails the impersonation of a device to alter data or processes [91]. Creating AI security systems might alleviate these vulnerabilities by guaranteeing the ethical and technological dependability of AI systems employed in IIoT, therefore diminishing the likelihood of application-level attacks. Techniques like micro-perturbations reveal concealed intruders by implementing little-controlled alterations to sensor readings, facilitating the identification of unlawful data without disrupting system operations [92]. It is essential to balance the utilization of AI for security enhancement with the management of related risks for the sustainable development of IIoT systems [93].

#### 3.3.2. Processing Layer Attacks

The processing layer of IIoT is susceptible to numerous threats that can jeopardize the security and functionality of IIoT systems. These assaults exploit weaknesses in the network, devices, and data processing systems, necessitating comprehensive detection and mitigation measures. The IoT-Defender technology integrates a modified genetic algorithm with a long short-term memory (LSTM) network to identify cyberattacks within IoT networks. This methodology enhances feature selection and model parameters, guaranteeing elevated detection accuracy and efficiency. It is engineered for seamless deployment on edge servers. AdaptSDN employs software-defined networking (SDN) and ensemble learning to safeguard IIoT applications in 6G environments, allocating network resources and segmenting devices to mitigate the effects of attacks, utilizing digital twins for real-time threat detection.

#### 3.3.3. Network Layer Attacks

Network-level attacks frequently entail intrusion attempts that may impair communication among devices. The network layer is especially vulnerable to intrusion attacks, such as DoS and DDoS attacks, which can inundate network resources and IIoT services by overwhelming the network with traffic [94]. Strategies like Temporary Dynamic IP Addressing (TDIP) efficiently thwart such assaults by constantly altering IP addresses, hence enhancing network security [95]. Malefactors can infiltrate IIoT devices and establish botnets, which are networks of compromised devices utilized for extensive assaults such as DDoS. These assaults can inundate IIoT systems, resulting in considerable downtime and operational malfunctions [96]. IIoT devices are susceptible to purposeful electromagnetic interference and eavesdropping, potentially leading to data breaches and service interruptions. Physical-layer security solutions can alleviate these concerns through cooperative transmission mechanisms to guarantee data availability and confidentiality [97]. 

#### 3.3.4. Physical (Perception) Layer Attacks

The perception layer of IIoT is essential for the collection and processing of data from many sensors and devices. Nonetheless, it is vulnerable to several forms of assaults that can jeopardize data integrity, confidentiality, and availability. Perception layer attacks focus on devices and equipment that engage with the physical environment, including sensors, actuators, controllers, and other devices within the perception layer. This layer, tasked with data collection, is susceptible due to the constrained computing power and storage capacity of the devices. Malefactors can capitalize on these constraints to introduce erroneous data or interfere with data collection procedures. Physical layer attacks entail the manipulation of hardware components within IIoT systems. The utilization of battery-powered IoT platforms for monitoring industrial equipment may be susceptible to physical manipulation, resulting in erroneous data collection and system malfunctions [98]. These attacks may lead to data falsification, unauthorized access, and additional security breaches, potentially resulting in severe repercussions for industrial systems. IoT systems including video are susceptible to motion-based video attacks. These attacks employ spatiotemporal attention networks to produce imperceptible perturbations that are challenging for human observers to discern [99]. In IoT-based smart grids, adversarial attacks can distort text data analyzed by natural language processing (NLP) technology. These attacks can modify sentence-level data, deceiving classification machines without substantially altering the semantic meaning [100]. The emergence of novel sensor platforms, like event-based multi-electrode arrays for biosignal detection, underscores the necessity for robust physical security protocols to avert illegal access and interference [101]. A framework based on Software-Defined Networking (SDN) utilizing machine learning techniques has been established for the surveillance of Industrial Internet of Things (IIoT) devices and networks. This framework employs SVM and decision tree models to accurately detect network intrusions and effectively safeguard the perception layer against unwanted access [102]. A hybrid technique utilizing deep neural networks (DNN) has been presented for the detection and classification of distributed denial-of-service (DDoS) attacks in Industrial Internet of Things (IIoT) networks. This approach employs XGBoost for feature selection and a CNN-LSTM model for classification, guaranteeing excellent accuracy and minimal latency, which is crucial for real-time IIoT applications [103].

**Table 2:** Cybersecurity of IIoT–edge computing.

Authors	Type of Attacks	Effects	Methods
[104]	Physical (perception) layer attacks: - Attacks on wireless IoT platforms, on videos, on images - Adversarial attacks	- Threat to data integrity, confidentiality, and availability - Authenticated intrusions - Inaccurate data collection and system failures - Unauthorized access and manipulation - Data modification at the statement level - Misleads classification models - Device failures - Data integrity violations and operational failures	- CNN-SRU improves data processing and accuracy - A bi-color encryption system combines two color images, confusing attackers and demonstrating resilience to known threats - Multi-electrode event-based matrix probes biosignals to prevent unauthorized access - SDN with ML, SVM, Decision Tree - XGBoost, and CNN-LSTM for real-time IIoT applications - BigRU and Inception-CNN enhance detection rates and address data imbalance - CANN - Image anomaly detection (IAD) - CNN-SRU
[105,106,107,108]	Network layer attacks: - DoS - DDoS - MiTM - Malware - Botnets - Interference and eavesdropping	- Attack devices - Disrupt communication between devices - Overload network resources and disrupt service availability - Downtimes and failures - Data leaks and service interruptions	- ML and DL models effectively detect and block network attacks with high accuracy - AES computational architectures enhance network security - Temporary dynamic IP addresses (TDIP) prevent DoS attacks - Reinforcement learning and physical level security (PLS) reduce interference and eavesdropping attacks
[109,110,111]	Processing layer attacks: - APT - Malware - Data Integrity Attacks - DDoS	- Intelligence data collection or operational disruption - Entry of inaccurate data affecting decision-making - Slowing or halting data processing - Release of defective products - Equipment damage - Operational management failures - Decision-making delays	- IoT-Defender optimizes features and model parameters for high accuracy and detection speed - IFTTT highlights potential attack pathways and entry points from device interactions - AdaptSDN protects IIoT applications in 6G by isolating devices and using digital twins for real-time threat detection
[112]	Application layer attacks: - Malware - Cascade attacks - Eavesdropping and spoofing	- Input of false data - Unauthorized data control and manipulation - Ransomware can encrypt critical data, halt operations, and demand ransoms - IIoT disruptions can impact supply chains, smart city security, and energy grids - Interaction between devices creates vulnerabilities - Data leaks and theft - Privacy violations - Interception and alteration of communications - Unauthorized access to confidential information - Data or operation manipulation	- Development of AI security systems - Micro-distortion methods can expose hidden attackers - BigRU and Inception-CNN significantly enhance IDS intrusion detection levels - AdaptSDN uses SDN to isolate IIoT devices into network segments, limiting attack impact and reducing cascade failures - IoT-Defender is effective in edge computing environments and improves attack detection

**Figure 5:**
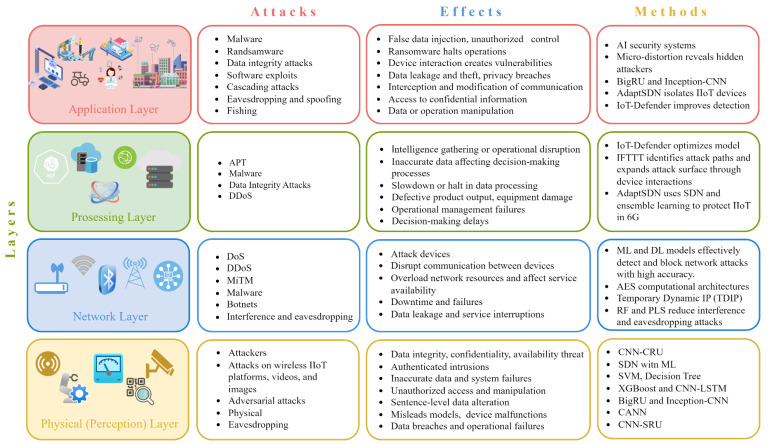
IIoT layers: common attacks, effects, and mitigation methods.

Figure 5 shows the different levels of IIoT, the common types of attacks, their impacts, and the protective measures for each level. Attacks include malware, data integrity issues, and ransomware at the application layer, along with DoS/DDoS attacks, interception, and eavesdropping at the network and physical layers. These attacks can result in significant issues, including device malfunctions, data leaks, interruptions in operational management, and even physical harm to equipment. To combat these threats, a range of strategies is employed, such as AI security systems, machine learning, software-defined networks (SDN), and sophisticated algorithms like XGBoost and CNN-LSTM. A thorough approach to safeguarding all aspects of IIoT is essential for ensuring system resilience against cyber threats and for maintaining seamless operations.

## 4. The Role of Edge Computing in the IIoT

This section explores the significance of edge computing within the IIoT landscape. It looks into how edge computing boosts IIoT performance and assesses the effectiveness of existing security measures.

### 4.1. Application of Edge Computing IIoT

The integration of IIoT with edge computing addresses the limitations of centralized systems, such as latency, security risks, and resource limitations. By processing data at its source, edge computing enables real-time decision-making, reducing network congestion and improving overall operational efficiency. This makes it an essential tool for enhancing industrial processes and protecting data. 

#### 4.1.1. Architecture

Edge computing is an architecture that facilitates the real-time collecting, processing, and analysis of data directly on manufacturing lines. Information from devices like sensors and actuators is relayed to control systems, which is essential for low-latency applications, such as intelligent transportation systems. The proximity of data processing to the source diminishes latency and enhances response times, which is crucial for the effective functioning of energy systems. The incorporation of AI and ML into edge devices facilitates real-time data processing and decision-making, diminishing the necessity for constant cloud connectivity [113]. Within the framework of smart city projects, edge computing integrates with AI and software-defined networking (SDN) to facilitate autonomous operations [114]. Edge gateways serve as mediators between devices and the cloud, enhancing computational capacity and optimizing bandwidth utilization, hence facilitating safe data transmission over the IIoT network [115]. 

The cloud layer offers computing resources and storage solutions for intricate data analytics and long-term administration, enhancing edge computing for applications that necessitate substantial computational capacity or lack stringent time limitations [116]. The cloud platform facilitates centralized data analysis and application management. The interaction between edge and cloud computing is enabled by microservices and service-oriented architectures, allowing for flexible resource integration. To improve learning efficiency, federated learning systems facilitate the distribution of jobs to edge servers, thereby decreasing latency and energy usage [117].

**Figure 6:**
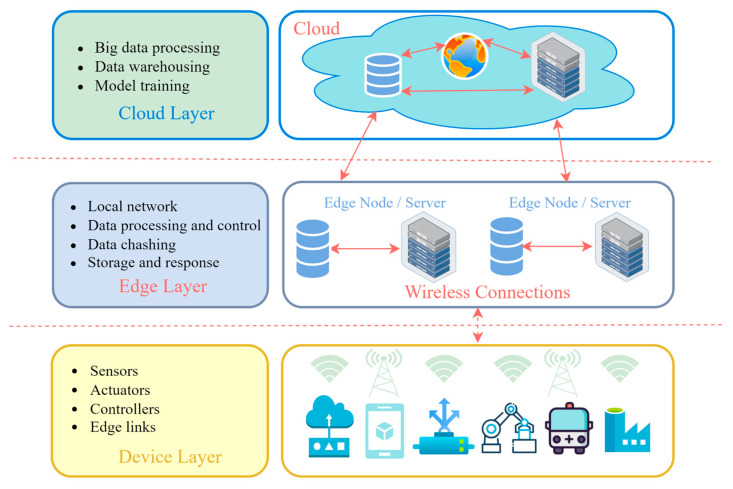
Architecture of IIoT–edge computing.

The IIoT–edge computing architecture plays a crucial role in achieving high performance and low latency for real-time applications by effectively integrating local and cloud data processing. Edge computing allows for data collection and processing on production lines through sensors and actuators, while edge gateways connect local devices to cloud services, improving the efficiency of network resources. The cloud layer provides storage and advanced analytics capabilities, enabling the creation of local digital twins and the implementation of artificial intelligence models. This integration creates a flexible system that meets the demands of modern production and urban projects (Figure 6).

#### 4.1.2. Applications

Edge computing enhances the diagnosis and prediction of industrial machinery through AI by processing data locally, which reduces the need for constant server connectivity. This approach allows for quick insights and timely evaluations of machine conditions [118]. In smart manufacturing, edge computing supports decentralized optimization through federated learning platforms, ensuring low latency and secure data processing. This is vital for handling various types of industrial data and improving manufacturing operations. Edge computing also enables real-time fault diagnosis and remote localization in industrial environments. By deploying lightweight models on edge devices, high accuracy and reliability are maintained, particularly in noisy settings [119].

#### 4.1.3. Benefits

Local data processing allows edge computing to significantly lower response times, which is critical for real-time applications in industrial environments. Additionally, edge computing minimizes data transmission to central servers, thereby enhancing data privacy and security.

#### 4.1.4. Security and Anomaly Detection

Security plays a vital role, and cutting-edge technologies like hybrid CNN+GRU deep learning models are employed to defend IIoT systems against cyber threats through anomaly detection. These models improve security by effectively spotting anomalies, which helps to protect against possible cyber threats.

#### 4.1.5. Challenges and Solutions

Edge devices frequently possess constrained processing resources, which may impede the implementation of sophisticated applications. Solutions like serverless edge computing and optimized workflow scheduling tackle these challenges via effective resource and dependency management [120]. Integrating edge computing in industrial settings presents challenges due to varied application needs and environmental limitations. Creating efficient algorithms and technology designed for certain applications can address these issues. 

**Figure 7:**
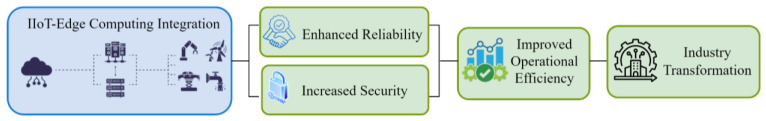
Importance of integrating IIoT technologies and edge computing.

Figure 7 illustrates the significance of integrating IIoT and edge computing technologies in order to convey the concept of Smart Industry. It emphasizes critical aspects of transformation in industrial processes via the utilization of linked devices, sensors, and automation systems. Consequently, edge computing and IIoT technologies fundamentally transform the management of industrial processes by delivering rapid data processing and facilitating real-time decision-making at the device level. These technologies diminish dependence on cloud solutions, decrease latency, and improve industrial adaptability. Integrating IIoT with edge computing enhances coordination, quality, and operational efficiency, generating new potential for adaptation to dynamic situations and optimization of the entire value chain. The implementation of these technologies is essential for enhancing reliability, security, and innovation in contemporary industry.

### 4.2. What Ways Can Edge Computing Enhance IIoT, and What Cybersecurity Benefits Does Its Implementation Offer?

Edge computing not only increases production efficiency but also markedly improves data security by processing information locally. This diminishes the volume of data transmitted to the cloud, hence mitigating the danger of data breaches or illegal access. Edge computing enhances IIoT by optimizing data processing efficiency and fortifying cybersecurity safeguards, enabling immediate data analysis and decision-making at the network edge, which is essential for industrial applications and necessitates swift response times.

#### 4.2.1. Real-Time Data Processing

Edge computing minimizes latency, enhances real-time analytical capabilities, and establishes a robust security framework by processing data nearer to the source, which is essential for the IIoT ecosystem. Edge computing facilitates immediate data analysis and self-directed decision-making, crucial for industrial applications that demand rapid reactions, including autonomous vehicles, smart city infrastructures, and industrial robotics [121]. Proximity in data processing reduces latency and bandwidth usage, essential for IIoT applications that demand prompt replies.

#### 4.2.2. Augmented Cybersecurity Protocols

Storing and processing data on local devices enhances both security and privacy. Edge computing improves IIoT security through decentralized processing and stringent standards, hence diminishing the danger of cyber threats and constraining the attack surface. Machine learning (ML) and deep learning (DL) techniques proficiently identify intrusions, attaining great precision in recognizing attacks such as distributed denial-of-service (DDoS) and man-in-the-middle (MITM) attacks [122]. Anomaly detection models, encompassing hybrid deep learning architectures, enhance security by precisely spotting unusual patterns and thwarting potential attacks. Nonetheless, integration encounters obstacles concerning security and compatibility among diverse devices [123].

#### 4.2.3. Real-Time Anomaly Detection and Security

Real-time anomaly detection is a crucial component of security in IoT and IIoT systems, facilitating the swift identification of suspicious actions and the mitigation of hazards. The integration of edge computing with federated learning enhances the effectiveness of anomaly detection while preserving data privacy without the need for centralized storage [124]. Federated learning mitigates the hazards of data breaches and facilitates adaptive systems, enhancing performance in dynamic contexts [125]. It facilitates the training of models on dispersed sensors without the need to exchange raw data, hence improving security [126]. Edge computing facilitates the implementation of AI models at the periphery, promoting instantaneous data acquisition and industrial process oversight.

#### 4.2.4. AI Integration for Improved Security

The integration of edge computing with artificial intelligence and machine learning enhances the security of the Industrial Internet of Things. AI-driven techniques enhance privacy and security, adeptly mitigating dangers and establishing dependable trust mechanisms. The application of AI and ML techniques, including deep learning models, improves intrusion detection systems (IDS). A 1D-CNN model attained an F1 score of 93.8% in the classification of network traffic, illustrating its efficacy in identifying IoT threats [127]. Hybrid deep learning models, exemplified by the CNN+GRU architecture, have demonstrated a high accuracy of 96.41% in anomaly detection, which is essential for ensuring the security of IIoT systems [128]. The integration of edge computing and AI provides scalable and adaptive security solutions to combat emerging threats in IIoT environments. AI-driven techniques proficiently mitigate security concerns, establishing a robust framework for privacy and security in IoT devices.

**Figure 8:**
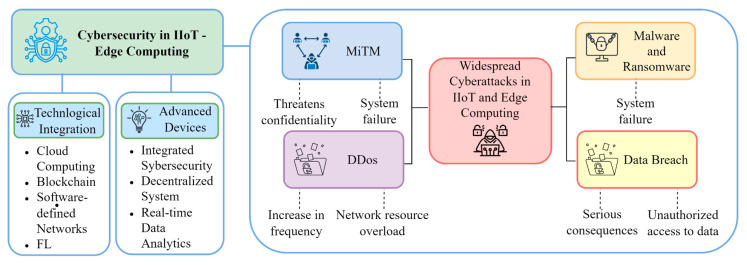
Cybersecurity challenges in IIoT–edge computing.

The IIoT and edge computing sectors have experienced an increase in the prevalence of attacks involving networks of compromised machines, data breaches, unauthorized access to computers and physical objects, malware, and ransomware in recent years, as demonstrated in Table 3. The aforementioned research underscores the importance of edge computing and machine learning in enhancing real-time data processing capabilities inside the Industrial Internet of Things (IIoT). The integration of technologies including cloud computing, blockchain, software-defined networking (SDN), and federated learning significantly diminishes latency and improves the efficiency of industrial systems. The developed algorithms provide great accuracy in attack detection and process optimization, as proven by their implementation in real-world industrial environments. Advanced devices with integrated cybersecurity and decentralized training systems proficiently mitigate DDoS attacks, man-in-the-middle attacks, and network threats, simultaneously enhancing security and performance in IIoT, as illustrated in Figure 8. 

### 4.3. Cybersecurity of Industrial Internet of Things–Fog Computing Systems

The security of the IIoT in fog computing systems presents a considerable challenge due to intrinsic vulnerabilities and complexities. IIoT systems that incorporate fog computing are vulnerable to interference, eavesdropping, and cyberattacks due to their decentralized architecture and dependence on wireless connectivity. To enhance the security of these systems, many frameworks and architectures have been proposed, emphasizing the physical layer, zero trust architectures, digital twin-managed platforms, blockchain-based solutions, and privacy-preserving audit mechanisms.

#### 4.3.1. Access Control and Resource Management

An effective access control system is crucial for safeguarding fog computing environments. The suggested system incorporates monitoring and risk assessment functionalities to identify anomalous user behaviors, hence averting illicit acts. The system employs a trusted access certificate for user identification and authorization, ensuring elevated security and data privacy while reducing latency.

#### 4.3.2. Deep Learning for Intrusion Detection

A lightweight and safe framework employing deep learning techniques, specifically artificial neural networks (ANNs), is suggested for the detection of security vulnerabilities in fog computing environments. This architecture observes network traffic and has exhibited superior accuracy in safeguarding against vulnerabilities, surpassing conventional methods such as SVM and decision trees. 

#### 4.3.3. Physical Layer Security

A collaborative architecture has been established to avert interference and eavesdropping assaults in IoT contexts. This method employs physical-layer security techniques to guarantee information accessibility and confidentiality. Through the implementation of cooperative data transmission, IoT devices can obstruct signal transmission for eavesdroppers, effectively averting unlawful access to sent data [130].

#### 4.3.4. Zero Trust Architecture

The zero trust security approach is implemented in IIoT platforms to avert illegal access and data breaches. A novel approach has been established utilizing fuzzy learning in conjunction with Streebog cryptographic substitution based on the SCSPN-TFL data transmission network, enhancing data integrity and diminishing false positives relative to current methodologies. A versatile zero trust architecture for IIoT infrastructures has been developed, integrating network micro-segmentation and software-defined networking (SDN). This architecture decentralizes operations while preserving centralized security policy administration, hence augmenting the robustness of IIoT systems against cyber threats [131].

#### 4.3.5. Digital Twin-Managed Security

A digital twin-managed platform using interactive ensemble machine learning to identify and mitigate real-time assaults can be utilized. This methodology tackles issues associated with extensive and uneven data in IIoT settings, delivering efficient attack detection akin to offline techniques [132].

#### 4.3.6. Blockchain-Enabled Security

The SecureArchi-IIoT operational architecture, founded on blockchain technology, improves the security and privacy of IIoT operations. It employs smart contracts and a reputation-based behavioral penalty mechanism to regulate operational permits and enhance security efficiency, exhibiting greater security and privacy than conventional systems [133].

#### 4.3.7. Privacy

A dynamic privacy-preserving audit scheme for IIoT systems utilizing cloud technologies has been created. This system employs regeneration storage through coding to improve data security, minimize latency, and guarantee resilience against diverse attacks while sustaining low computational expenses. 

## 5. Integration of CPS with IIoT

This section analyzes the importance of CPS in IIoT, highlighting their robustness, security, versatility across multiple domains, and new problems.

### 5.1. Cyber-Physical Systems and Their Significance in Industrial Internet of Things

CPS are essential in the IIoT because they integrate computational and physical processes to improve operational capabilities, security, and efficiency across industries. These systems are vital for real-time data acquisition, processing, and analysis, which is crucial for the uninterrupted functioning of IIoT applications. The significance of CPS in IIoT is further emphasized by developments in sensor technology, data protection, and AI-driven decision-making. The incorporation of AI into sensors facilitates the identification and mitigation of performance decline, promoting innovation and enhancing industrial operations [134]. Machine learning (ML) is essential in network security, executing functions such as anomaly detection and adaptive threat response, hence enhancing the resilience of IIoT systems [135].

Blockchain technology integrated with AI offers a formidable solution for safeguarding cyber-physical systems (CPS) from advanced attacks, guaranteeing secure data transfer and storage, while bolstering confidence among stakeholders and enabling predictive maintenance [136]. The application of AI in CPS facilitates the creation of intelligent systems that can independently manage and optimize industrial operations. Notwithstanding substantial advancements in cyber-physical systems for the Industrial Internet of Things, difficulties including cybersecurity, privacy, and compatibility persist [137]. The use of cryptography with identifiers in CPS fulfills privacy and data integrity needs, essential for IIoT applications. This approach guarantees safe data transmission while facilitating open search capabilities, hence enhancing operational efficiency [138].

### 5.2. Application

CPS enhances smart grids by the integration of contemporary management, monitoring, and communication technologies to provide a consistent power supply, hence increasing the efficiency of generators and distributors [139]. Within the framework of Industry 4.0, cyber-physical systems (CPS) are pivotal in revolutionizing robotics and machine control systems through the use of artificial intelligence (AI) and machine learning (ML). These technologies improve efficiency and accuracy while decreasing energy usage and optimizing operations [140]. CPS also contribute to the formulation of models for resource allocation in motion control systems, ensuring the efficient management of computing resources and processes utilizing diverse CPU elements and communication interfaces.

### 5.3. Emerging Challenges

Despite the various advantages of CPS, difficulties such as cyber threats and data privacy persist as substantial obstacles that must be resolved to fully realize their potential in IIoT environments. Cyberattacks against cyber-physical systems (CPS) can result in substantial operational interruptions, particularly in industrial environments where CPS governs essential infrastructure. Such disruptions may impede production lines, impact supply chains, and lead to significant downtime. The financial repercussions of cyberattacks on CPS can be significant, encompassing direct expenses from operational interruptions and indirect costs stemming from reputational harm. While CPS markedly enhance the functionality and security of IIoT systems, they concurrently add complexities pertaining to data privacy, security, and trust. Mitigating these obstacles via stringent security protocols and transparent AI systems is essential for maximizing the promise of CPS in IIoT applications. Cyber-physical systems encounter challenges including security, trust management, and compatibility, which are essential for their effective deployment. Ongoing progress in CPS technologies fosters creative enhancements in efficiency and resource management.

The studies presented in Table 4 emphasize the crucial role of sensors and AI in boosting performance and safeguarding data. By combining blockchain with AI, threats can be effectively reduced, and cryptography is essential for maintaining security. Major challenges such as trust management, cyberattacks, and data privacy need additional attention to enhance the resilience of these systems.

**Figure 9:**
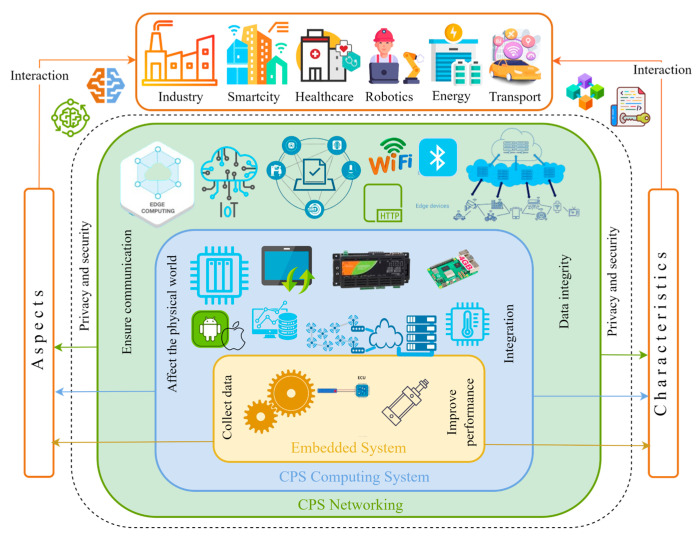
CPS aspects and technologies in IIoT.

Figure 9 shows the structure and important features of CPS technologies in the IIoT landscape. Cyber-physical systems play a crucial role in connecting the physical world with digital technologies, enabling various industrial sectors to improve their operations and automation through IIoT. Embedded systems gather data from physical devices, while the CPS computing system analyzes these data and makes informed decisions. CPS networking facilitates communication among system components and allows for data transmission using wireless technologies like WiFi, Bluetooth, and various communication protocols. Key challenges for CPS include privacy, security, connectivity, and their effects on the physical environment. Additionally, maintaining data integrity and enhancing performance are vital for the successful operation of these systems. The image illustrates how CPS integrates physical devices with digital technologies to develop intelligent and secure management systems across different industries, highlighting essential aspects such as data security, performance, and integration.

### 5.4. Overview of Existing Attacks in CPS IIoT

The landscape of cyberattacks in IIoT and CPS is continuously changing due to the adoption of advanced technologies, which makes these systems more susceptible to threats. While operational efficiency has improved across various sectors, these advancements also bring about considerable security risks. The repercussions of attacks can be severe, ranging from operational disruptions to risks to human safety and privacy. Figure 7 illustrates the most prevalent types of attacks on IIoT and CPS and their associated impacts. 

DoS attacks represent a major risk to CPS, particularly those utilizing the TCP protocol. Such attacks can destabilize systems by interrupting communications, potentially leading to operational failures. When CPS is modeled under the influence of DoS attacks, it is treated as a system with switching linear parameters, underscoring the necessity for effective management strategies to ensure stability. DoS attacks also seek to disrupt service availability by bombarding the system with excessive traffic, which poses a significant threat in IIoT environments where uninterrupted operation is essential. 

Man-in-the-middle (MITM) attacks enable attackers to intercept communications between devices, with the potential to alter or steal sensitive information, thus posing a serious risk to IIoT. Eavesdropping and IP spoofing are linked to unauthorized data access and device impersonation, compromising the confidentiality and authenticity of communications [141]. Enhancing authentication processes can help to prevent unauthorized access and mitigate the risks of identity spoofing. Data injection involves the introduction of false data into the system, which can result in incorrect decisions and operational failures in CPS. Replay attacks involve intercepting and retransmitting valid data to mislead the system, impacting the integrity and reliability of CPS operations [142]. In energy systems, attackers can exploit controllers using deep reinforcement learning (DRL) by introducing minor disturbances, making these attacks challenging to detect and potentially having a significant impact. DRL shows greater resilience against attacks, and by incorporating robust learning methods, we can further mitigate the effects of these attacks on DRL controllers. This enhancement boosts their capacity to endure disturbances and improves overall system security [143]. Real-time monitoring systems in energy sectors utilize temporal logic signals to detect and pinpoint anomalies. These systems scrutinize network data to spot deviations from expected behavior, thereby lowering the risk of cyber threats in smart grid substations and SCADA systems [144]. IoT devices face risks from traffic analysis attacks, where attackers can glean sensitive information from network traffic. Even with existing safeguards, emerging attack models, like image-based attacks, can bypass these defenses, highlighting the urgent need for better privacy protection strategies. 

These studies underscore the vulnerabilities and repercussions of cyberattacks on IIoT and CPS, stressing the necessity for effective protective measures. While current strategies (Table 5) lay a strong groundwork, the evolving nature of threats demands continuous adaptation and innovation, and intelligent control methods will enhance the security of IIoT and CPS.

Cyberattacks targeting cyber-physical systems represent a serious risk to industrial infrastructure in today’s digital landscape, particularly with the rise of the Industrial Internet of Things (IIoT). Threats like Denial of Service (DoS), man-in-the-middle (MiTM) attacks, and traffic analysis can severely disrupt industrial operations by undermining the confidentiality, integrity, and availability of critical data. It is crucial to not only recognize the potential impacts of these attacks but also to formulate effective risk mitigation strategies. Tackling cybersecurity issues demands a holistic approach, which includes measures such as network segmentation, stronger authentication protocols, and the adoption of AI-driven solutions, including deep learning techniques. These strategies are vital for preventing attacks and reducing their effects on essential processes, particularly in sectors like energy, transportation, and manufacturing. A multi-layered defense strategy that combines both technical and organizational tactics is essential for effectively addressing contemporary threats and ensuring the resilience of industrial operations. This highlights the necessity of a thorough approach to safeguarding against cyberattacks.

### 5.5. CPS Cybersecurity

Cybersecurity in CPS within the IIoT is crucial because it combines physical processes with computational capabilities. While this integration boosts efficiency, it also presents considerable security challenges.

Various models have been suggested that focus on vulnerability assessment, data categorization, and cost–benefit countermeasures to safeguard IIoT from cyber threats [145]. One such method, known as identification-based encryption (IBSC-PCE), has been created to ensure the secure transmission and retrieval of encrypted data, outperforming existing techniques [146]. Additionally, a deep learning-based threat identification mechanism has been developed to effectively detect attacks on industrial control systems (ICS) [147]. Digital forensics tools (DFIR) are also instrumental in maintaining data confidentiality and providing reliable intrusion detection [148]. It is vital to integrate security measures like access control and authentication for the effective operation of CPS in IoT.

Secure frameworks such as AdaptSDN utilize software-defined networking (SDN) and ensemble learning to enable dynamic resource allocation and enhance intrusion detection, thereby ensuring the reliability of IIoT applications in 6G networks. Anomaly detection models that employ deep learning techniques are essential for identifying and addressing security threats in the IIoT landscape [149].

Despite notable advancements in IIoT systems, these technologies bring forth new challenges, including expanded attack surfaces and privacy concerns. Tackling these issues necessitates ongoing innovation and continuous research to adapt to emerging threats, ensuring security and fostering the development of adaptive learning methods for the safe and efficient operation of IIoT networks.

**Figure 10:**
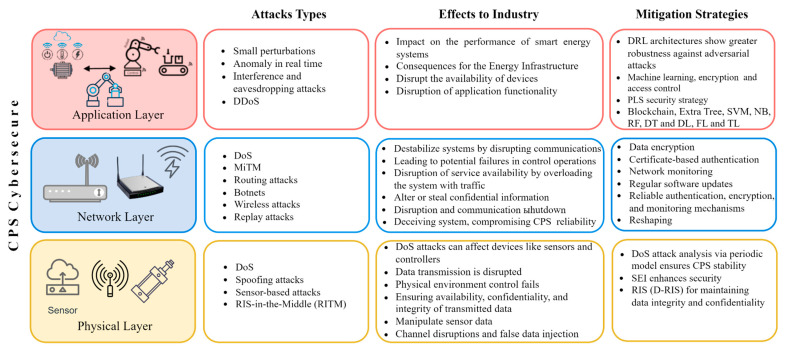
Types of cyber attacks on CPS and their impact on industry.

A multi-layered structure of cyber threats and protection methods, illustrated in Figure 10, outlines the security challenges faced by CPS in IIoT. Each layer presents its own vulnerabilities that need to be addressed when creating robust architectures. At the application level, threats like DDoS attacks and data interception reveal the susceptibility of CPS to failures, which can degrade system performance. To maintain the availability of IIoT applications in real-time, it is essential to implement mechanisms such as machine learning for anomaly detection and blockchain for ensuring data integrity. The network level is vulnerable to MiTM and routing attacks, making encryption and active monitoring vital. Regular software updates and traffic analysis are necessary to adapt to emerging threats. At the physical level, CPS components, including sensors, face risks from DoS attacks and data falsification, which can compromise the accuracy of information gathering. Strategies like RIS-in-the-Middle (RITM) and DoS attack analysis can help to reduce these risks. The interdependencies across layers necessitate that protective measures at one level do not compromise others; for instance, the heavy load from blockchain can lead to operational delays. Future research should aim at integrated solutions that strike a balance between performance and security. In summary, a holistic approach to CPS security is increasingly vital. Adaptive and intelligent solutions are crucial for safeguarding infrastructure against intricate cyber threats, ensuring the resilience and reliability of IIoT systems.

## 6. Methods for Enhancing the Security of IIoT Cyber-Physical Systems Using Edge Computing

This section explores various strategies to bolster the security of IIoT cyber-physical systems through the use of edge computing. Given the increasing prevalence of cyber threats and the imperative to safeguard critical infrastructure, there is a strong focus on incorporating edge computing capabilities to enhance both data and system security.

### 6.1. AI Methods

The strategies for improving the security of IIoT cyber-physical systems with edge computing utilize its capabilities to facilitate real-time data processing, minimize latency, and strengthen security protocols. By combining edge computing with cutting-edge technologies like digital twins, blockchain, machine learning, and cryptographic techniques, a solid framework for safeguarding IIoT systems is established. AI methods are especially useful for detecting anomalies in real-time, allowing for the swift identification and resolution of potential issues in industrial operations.

The combination of blockchain and edge computing greatly improves the security of IIoT cyber-physical systems by tackling issues related to data privacy, intrusion detection, and resource limitations. The AI-based lightweight blockchain security model (AILBSM) enhances IIoT security by integrating blockchain technology with artificial intelligence techniques, such as the optimized Sprinter Convivial Neural Network (COSNN). This model converts functions into encoded data, significantly improving anomaly detection and reducing execution time. When blockchain is paired with federated learning, it creates a strong foundation for intrusion detection in IIoT systems. Federated learning decentralizes the training process, allowing IIoT devices to collaboratively develop models without sharing raw data, thus maintaining privacy. Blockchain adds an extra layer of security by verifying the integrity of shared parameters, preventing tampering, and ensuring that only trustworthy data contribute to the global model [150]. The integration of blockchain with federated learning has been proven to significantly improve intrusion detection accuracy, even in attack scenarios such as MITM attacks, by safeguarding the integrity and confidentiality of the data used for model training.

#### Federated Learning

Federated learning (FL) is being integrated into edge computing to facilitate secure data sharing among IIoT devices. The SecureIoT platform employs FL for federated learning, which helps to achieve high accuracy in detecting attacks. This method effectively tackles privacy and security concerns while enabling efficient and secure data exchange between cyber-physical systems [151]. The blockchain-managed Border Intelligence (BoEI) platform uses decentralized federated learning for detecting cyberattacks. The Fed-Trust model incorporates a temporal convolutional generative network for semi-supervised learning, which enhances security and privacy through a reputation-based blockchain. This framework boosts both computational and communication performance, as shown by simulations conducted on publicly available datasets [152]. 

### 6.2. Integration of IT/OT Security

The integration of IT/OT security with edge computing takes advantage of the strengths of both IT and OT domains, creating a solid foundation to tackle the specific security challenges faced in IIoT environments. This integration enhances the security of IIoT cyber-physical systems. By combining IT and OT security in edge manufacturing networks that are supported by cloud technologies, a scalable and secure environment is established. This strategy aims to provide network-level security, validated against recognized threats and standards like IEC 62443-3-3 [153], and can be expanded to other levels for a more comprehensive security approach [154].

### 6.3. Intrusion Detection Systems (IDS)

IDS are essential for protecting IIoT and edge computing environments, which face increasing risks from sophisticated cyber threats. Various strategies have been developed to improve the effectiveness of IDS by tackling issues like high-dimensional data, imbalanced datasets, and limited resources. A holistic approach that combines Extra Tree (E-Tree), Deep Neural Networks (DNN), and Random Forest (RF) allows for effective intrusion detection through the analysis of IoT data traffic. This method has shown better performance in accuracy and stability when compared to traditional machine learning techniques [155].

### 6.4. Cryptography and Encryption

ML platforms based on blockchain technology improve the security and precision of edge services in IIoT. By leveraging smart contracts and public key cryptosystems, this framework guarantees data confidentiality and integrity while enabling efficient data processing and model accuracy. Public key cryptosystems offer a scalable solution for securing communication in IIoT settings, where many interconnected devices need to exchange data safely.

To safeguard IIoT operations, a framework has been introduced that uses lightweight encryption methods, along with modified ElGamal encryption and digital signatures. This strategy ensures secure and confidential data transmission, storage, and computation, surpassing existing models in terms of time complexity, latency, and energy efficiency. By implementing modified ElGamal encryption, the platform preserves data confidentiality without imposing significant computational demands, which is crucial in resource-limited edge computing environments. The incorporation of digital signature schemes within the SPTM-EC structure boosts data integrity and authenticity, ensuring that the data and tasks processed at the edge are not only confidential but also verifiable, thus preventing unauthorized access and tampering. The role of digital signatures complements encryption by providing additional security.

### 6.5. Edge Computing

The IoT-Defender framework utilizes a modified genetic algorithm (MGA) alongside a long short-term memory (LSTM) network to detect cyberattacks. This method improves both feature selection and model parameters, leading to better detection accuracy and efficiency [156]. The framework is designed to be lightweight, making it ideal for deployment on edge servers, and it supports real-time threat detection. By analyzing data closer to its source, edge computing minimizes latency and reduces bandwidth usage, which is essential for real-time applications. This decentralized strategy also bolsters data privacy, as sensitive information does not need to be sent to centralized cloud servers.

**Figure 11:**
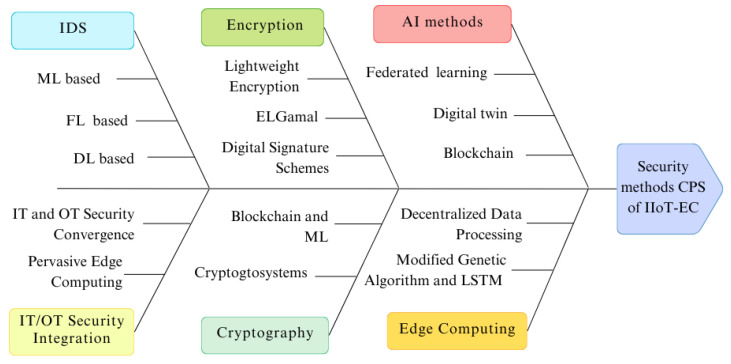
Security methods in CPS of IIoT with integration edge computing.

In summary, the use of security techniques in cyber-physical systems within the context of EC-IIoT—such as the integration of IT and OT security, machine learning (ML), deep learning (DL), federated learning (FL), blockchain, edge computing, ML-, FL-, DL-based intrusion detection systems (IDS), and cryptography—showcases a diverse range of technologies aimed at improving the reliability and security of industrial systems (Figure 11).

## 7. Discussion and Recommendation

### 7.1. IIoT Attacks

Cyberattacks are on the rise worldwide, with a notable increase in reported incidents seen in 2023–2024 [157]. The ENISA [157] report highlights eight major types of threats, with ransomware, DDoS attacks, malware, data threats, information manipulation, and supply chain attacks being the most prevalent. The data on IIoT-level attacks shows threats that can severely impact the critical components of industrial systems. Ransomware, DDoS, supply chain attacks, and man-in-the-middle (MitM) attacks pose significant risks to the operation and security of these systems, affecting everything from data perception to network and application layers. The repercussions can be severe, including halted production processes, disrupted smart grid operations, exposed software vulnerabilities, and manipulated physical processes. Real-world incidents, like the Colonial Pipeline attack and the SolarWinds vulnerabilities, highlight the urgent need to secure all levels of IIoT systems to avoid substantial financial and operational setbacks (Table 6).

Thus, implementing cybersecurity measures across all levels of the Industrial Internet of Things is crucial for ensuring the stability and reliability of industrial operations.

Future research should prioritize the development of stronger security systems and the investigation of new technologies, like blockchain, to improve the security and privacy of the Industrial Internet of Things (IIoT). While edge computing enhances the efficiency and security of IIoT, it also introduces challenges such as limited resources and the necessity for scalable solutions. Future research should delve into advanced hybrid architectures and the integration of artificial intelligence to further bolster the security and performance of IIoT systems. 

**Figure 12:**
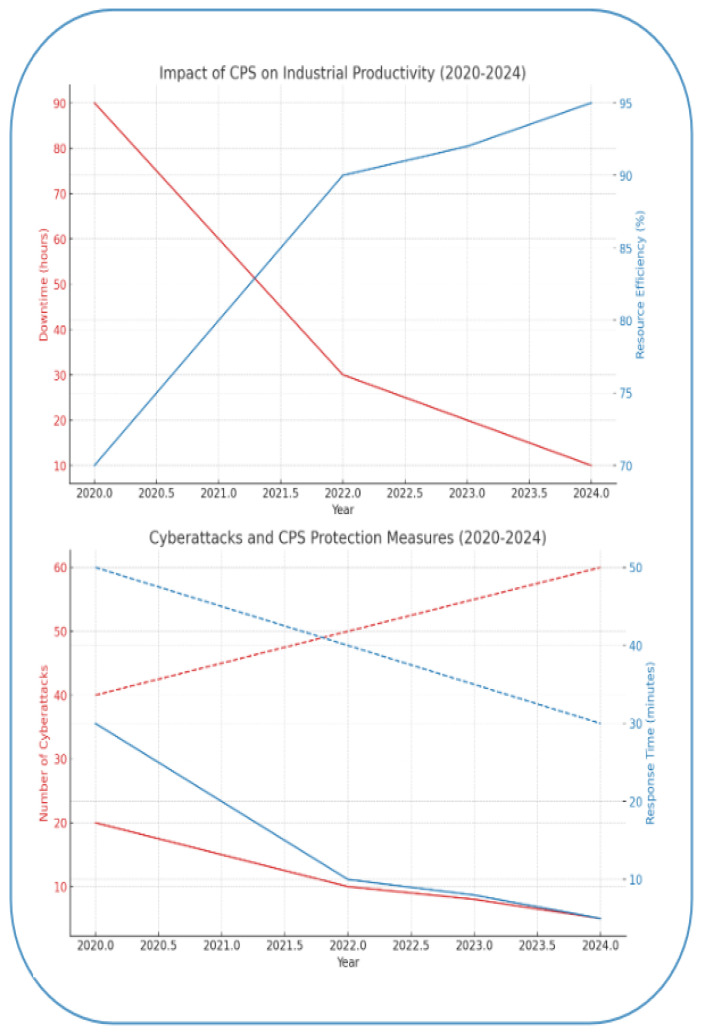
Impact of CPS on performance and cybersecurity in industry. The blue line in the top panel shows resource utilization efficiency increase, the red line is downtime. The straight blue line in the bottom panel shows the annual number of cyberattacks decreased, the red dashed line response time.

Figure 12 illustrates the considerable influence of cyber-physical systems (CPS) on both performance and cybersecurity within the industry from 2020 to 2024. After CPS implementation, downtime was reduced from 90 h to just 10, while resource utilization efficiency saw an increase from 70% to 95%. This reflects significant process optimization achieved through the use of AI and predictive analytics. At the same time, the annual number of cyberattacks dropped from 40 to 5, and response times improved dramatically from 50 min to just 5 min. These findings highlight the effectiveness of AI and blockchain technologies in safeguarding CPS. Overall, CPS not only boost performance but also greatly enhance the security of industrial systems.

### 7.2. Types of Cyber Attacks Against CPS

Attacks on CPS are increasingly likely to succeed due to their combination of physical and digital components. These vulnerabilities can result in disruptions to critical infrastructure and major operational failures. Cyberattacks like DoS, DDoS, zero-day exploits, and advanced persistent threats present significant security challenges for complex CPS infrastructures. Significant advancements have been made in creating new methods and strategies to prevent or detect cyberattacks, leading to enhanced security for cyber-physical systems [158]. A new model has been introduced to tackle DoS, replay, and spoofing attacks, incorporating forms that improve degradation and can be either state-dependent or independent [159]. AI-based tools have been employed to automate penetration testing in IT systems, mitigating the effects of attacks in operational technology environments [160,161]. Table 7 provides a detailed overview of cyberattacks on CPS and the defense methods.

Table 7 demonstrates the advantage of AI methods in detecting cyberattacks on CPS.

### 7.3. Analysis of Cybersecurity Approaches for Cyber-Physical Systems in IIoT–Edge Computing Integration

Various AI techniques, blockchain, edge computing, IT/OT integration, encryption, and cryptographic methods—including lightweight encryption and digital signatures—offer data protection at the communication level. Enhanced AI-driven strategies and modified algorithms improve threat detection and the overall efficiency of IIoT cyber-physical systems. The incorporation of network monitoring solutions and intrusion detection systems (IDS) that utilize machine learning (ML), deep learning (DL), and federated learning (FL) also aids in the swift identification of anomalies and real-time threat responses. Consequently, these strategies establish a multi-layered security framework that bolsters the resilience of IIoT against cyberattacks and reduces the risks of disruptions to critical infrastructure. 

Table 8 provides a comparison of our approach with other studies.

The IIoT architecture encounters several security challenges that jeopardize the integrity of cyber-physical systems. Key vulnerabilities consist of weak authentication, poor encryption, and software flaws, which leave systems open to attacks like Denial of Service and man-in-the-middle. These weaknesses can undermine device integrity and the overall security of the system. Prevalent risks include unauthorized access stemming from insufficient authentication, data interception due to inadequate encryption [175], system failures arising from software issues, DoS attacks [176], routing attacks, and radio frequency interference [177]. Nonetheless, AI and blockchain technologies possess the capability to mitigate these vulnerabilitie.

The combination of FL, ML, and blockchain greatly improves the security of cyber-physical systems within Industrial Internet of Things and edge computing settings. This approach tackles privacy concerns, enhances intrusion detection, and minimizes risks linked to centralized systems. Federated learning (FL) facilitates model training on local devices, preserving data privacy and reducing potential failure points. Blockchain guarantees data integrity and offers a decentralized structure, which improves its ability to withstand attacks [178,179]. The combination of federated learning and blockchain boosts the development of effective intrusion detection systems. However, implementing these technologies presents obstacles, such as the substantial resource requirements of blockchain [180], the vulnerability of FL to hostile attacks like MITM, and the increased complexity of the system. 

An extensive overview of current research in the field of cybersecurity for CPS in IIoT and edge computing has been presented, highlighting essential technologies and solutions such as ML, FL, DL, blockchain, cryptography, and IDS. The contributions from various authors showcase a range of strategies aimed at tackling security and performance challenges in IIoT settings. These include the use of elliptic curve cryptography, resource optimization through edge computing, the incorporation of artificial intelligence for diagnostics and forecasting, and innovative solutions for securing Machine-to-Machine (M2M) communication while ensuring data privacy. Prominent research trends involve leveraging FL and blockchain to bolster security and privacy, alongside the deployment of new technologies to enhance attack detection and data management. It is crucial to recognize that merging edge computing with IIoT technologies can significantly boost operational efficiency, scalability, and the safeguarding of industrial systems.

The following are key gaps in current cybersecurity research for CPS in IIoT–edge computing:

- Integration of Cybersecurity and Physical Security—research frequently emphasizes cyberspace, neglecting the physical dimensions of CPS, which may result in vulnerabilities; a collaborative monitoring strategy for both domains can improve high detection accuracy [181].
- Administration of Diverse Devices—contemporary solutions frequently do not satisfy the demands of IIoT devices, including real-time functionality and decentralization; implementing a zero trust architecture with network micro-segmentation can enhance management.
- Identification of Uncommon Cyberattacks—the issue is intensified by the data imbalance in training datasets; advanced models, such as focal causal networks can proficiently rectify these imbalances, improving detection time [182].

## 8. Conclusions

This article provides a detailed overview of the current challenges and solutions in cybersecurity for cyber-physical systems (CPS) within the context of the Industrial Internet of Things (IIoT) and edge computing. It identifies common vulnerabilities and threats at different levels of the IIoT architecture, including denial-of-service (DoS) attacks, ransomware, malware, and man-in-the-middle (MITM) attacks, underscoring the importance of integrating edge computing to bolster CPS security. This research highlights that advanced technologies like machine learning, federated learning, blockchain, and intrusion detection systems (IDS) are essential for data protection and real-time attack detection.

The established taxonomy of security methods, along with a comparative analysis with other studies, underscores the relevance and effectiveness of the proposed strategies for safeguarding CPS amid the growing complexity and scalability of IIoT systems. Nonetheless, persistent challenges related to privacy and scalability remain, indicating a need for further research and the creation of new solutions to enhance resilience against cyberattacks.

Future research will aim to deepen the integration of IIoT with edge computing. A specialized service model for IIoT will be developed to manage cyber-physical incidents in transportation infrastructure, with the goal of reducing the impact of attacks on IIoT systems through the application of edge computing principles.

## Figures and Tables

**Table 1:** Current state of research in IIoT.

Authors	Year	Results	Key Issues	Not Considered
[51]	2022	IDS for protecting industrial CPS reaching 98.45% accuracy	Integration of AI, edge computing, enhanced security measures, 5G, and digital twins	Long-term sustainability, human factors, security cost analysis, and the impact of emerging technologies like blockchain
[52]	2023	CPS architecture for IIoT security. Protecting devices from cyber threats and improving detection accuracy with neural networks	IIoT faces challenges like heavy traffic, diverse networks, and high computing demands	The impact of new technologies on IIoT security
[53]	2024	The potential of smart factories in enhancing the manufacturing sector	CPS integrates technologies, upgrades systems, and ensures data security	Cybersecurity measures
[54]	2023	Implementation of blockchain and edge in IIoT	Integration of RCL, REL, and cloud computing layers	Blockchain architectures
[55]	2024	IDS for detecting attacks in IIoT using digital twin and online learning	Detecting attacks	Scalability and adaptability
[56]	2024	Intelligent intrusion detection system using SVD and SMOTE to improve accuracy	Modern IDS have flaws that intelligent recognition methods can address	Attack types
[57]	2024	IIoT across various industries and the importance of Edge AI for digital connectivity	The need to improve digital connectivity in IIoT using Edge AI	Problems of digital connectivity through Edge AI
[58]	2024	Anonymous authentication protocol for IIoT users, effective against attacks	Design of secure protocols to ensure security in IIoT	Security issues due to open wireless networks
[59]	2024	Security and architecture of distributed digital twins for maintenance	Digital twins: limited standards need better implementation and feedback	Feedback mechanisms
[60]	2023	Key IIoT security issues: attacks, data breaches, and the importance of encryption	Malicious attacks and privacy concerns in IIoT	Comprehensive solutions of security and privacy
[61]	2024	Role of AI in detecting and preventing cyberattacks in IIoT, essential for enhancing cybersecurity through ML, behavioral analysis, and NLP for anomaly detection	Data quality limits, high development costs	User experience and usability in cybersecurity solutions

**Table 3:** Role of edge computing in IIoT.

Authors	Year	Real-Time Data Processing	Cybersecurity	Attacks	AI	Results
[129]	2021	Data generation, architecture: cloud computing, network function visualization, blockchain, SDN, edge computing, and IoT/IIoT perception layers	-	DDos MITM information gathering malware	DT RF SVM KNN DNN	DNN achieves 94.67% for 15 classes and 96.01% for 6 classes, while DT scores 67.11% for 15 classes and 77.90% for 6 classes
[127]	2023	- Real-time data processing - Edge analytics, data filtering, and compression methods to minimize data volume and enhance application efficiency	Security protocols that reduce data transmission risks to cloud servers and improve overall IoT application security	-	-	-
[125]	2024	- Enhancing IIoT security with IDS that uses ML and DL for real-time threat detection - The system detects attacks in real-time, enabling rapid threat response	Secure IDS	Malware DDoS MitM	kNN DT	100%
[121]	2024	EC-IoT enhances real-time data processing by reducing latency and improving response time	Strategies for enhancing data and network security through the combination of EC-IoT and AI	-	-	-
[128]	2024	The Local Digital Twin (LDT) architecture at the edge enables real-time control.	-	-	ML	LDT on the assembly line in Brazil, using ML, improved productivity by 1.3–2.5%
[117]	2024	The federated platform enables fast data processing, with ADMM algorithms cutting response time by up to 58%	The decentralized system secures data and, with IIoT, reduces latency and boosts security	-	ML FL	Edge algorithms reduced response time by 17.2% and 58%, maintaining accuracy. Testing on power plant data confirmed the effectiveness of FL
[128]	2024	Real-time data processing is crucial for applications requiring timely decision-making	ML and FL methods assist in detecting cyber threats	-	ML FL	- KDE offers smooth, informative data representation - RNN learns complex sensor data relationships - ANFIS

**Table 4:** Comparative analysis of key aspects, technologies, and challenges in IIoT and CPS integration from last year.

Aspects	Categories
Sensors and AI	Improve performance
	State indicators ensure data integrity
Security and Integrity	Integration of heterogeneous networks and privacy protection
	Blockchain + AI = data protection from threats
	Prevents critical malfunctions data integrity
	Cryptography
Key Technologies	Sensors
	Machine learning
	Blockchain, AI
Applications	Motion control, resource distribution
	Industry, robotics
	Agriculture, healthcare
	Smart grids, energy systems
	Network security
	Transport systems, smart grids
Challenges	Trust management, secure routing protocols, integration of heterogeneous networks, and privacy protection
	Cyberattacks
	Cybersecurity, privacy, compatibility
	Process control
	Cybersecurity threats, data privacy

**Table 5:** Types of cyber attacks on CPS and their impact on industrial systems.

Types of Attack	Effects to Industry	Mitigation Strategies
DoS	Destabilize systems by disrupting communications, leading to potential failures in control operations. Disruption of service availability by overloading the system with traffic	Network segmentation and access control limit the spread of attacks and prevent unauthorized access to critical system components
MiTM	Alter or steal confidential information	Strengthening authentication can prevent unauthorized access and reduce identity spoofing risks
Replay attacks	Deceiving the system, affecting the integrity and reliability of CPS operations	Reshaping will change traffic patterns, making it harder for adversaries to access sensitive user information and ultimately improving user privacy
Small perturbations	Impact on the performance of smart energy systems	Deep reinforcement learning (DRL) architectures exhibit greater robustness against adversarial attack
Traffic analysis attacks	Extract confidential information from network traffic	The development of a traffic reshaping method that could significantly prevent image-based attacks aimed at IoT traffic analysis
Eavesdropping and IP spoofing	Confidentiality and authenticity of communications	-
Anomaly in real time	Consequences for the energy infrastructure	
Interference and eavesdropping attacks	Disrupt the availability of Internet of Things (IoT) devices	PLS strategy
DoS, DDoS	Disruption of application functionality	Blockchain, Extra Tree, SVM, NB, RF, DT and DL, and FL and transfer learning
Spoofing attacks	Ensuring availability, confidentiality, and integrity of transmitted data	SEI (Specific Emitter Identification) enhances security
Sensor-based attacks	Manipulate sensor data	-
RIS-in-the-Middle (RITM)	Channel disruptions and false data injection	RIS (D-RIS) by using non-cooperative communication channels and maintaining data integrity and confidentiality

**Table 6:** Attack effect examples on IIoT layers (2020–2024).

Attack Type	Affected Layers	Effect	Example
Ransomware	Perception, Application, Data	Production halt, financial loss, reputation damage	Colonial Pipeline (2021)
DoS	Network, Application	Disruption of real-time monitoring, operational downtime DDoS attacks can halt production lines, causing significant downtime and financial losses	Smart grid outages A DDoS attack on an intelligent manufacturing system can disrupt the entire supply chain
Supply Chain Attacks	All layers	Long-term, hidden vulnerabilities in software/hardware	SolarWinds (2020)
Firmware Exploits	Perception, Application	Physical process manipulation, production shutdowns	Siemens PLC vulnerabilities
MitM	Network, Perception	Data alteration, faulty operations	Smart manufacturing data tampering

**Table 7:** Cyber attacks against CPS and detection techniques.

Authors	Attack Type	Detection Techniques	Detection Accuracy
[162]	DoS	Kalman Filter	90%
[163]	FDIA	Sliding Mode Observer Methods	100%
[164]	DoS	Watermarking	100%
[165]	Intrusion	FL, GRU, RF	99%
[166]	Malware, password, phishing, SQL injection	Dynamic Estimator-Based Cyberattack Tolerant Control	99%
[167]	Generic	A Hybrid Deep Random Neural Network	98%, 99%
[168]	Evasion, data poisoning	RF, ANN, LTSM	96%, 98%
[169]	Deception attacks	Markov Chain	-
[170]	DDoS, password, backdoor, SQL injection, ransomeware, port-scanning, uploading, vulnerability scanner	LR, RF, CNN, SVM, kNN	100%

**Table 8:** Comparison of papers.

Research Field	Authors	Year	Contributions	Cybersecurity Methods				
				ML DL FL	Blockchain	IDS	IT/ OT	Cryptography and Encryption
IIoT CPS	[19]	2021	M2M communication in IIoT leverages advanced models like 5G, TSN Ethernet, and autonomous networks to improve manufacturing efficiency. It also tackles cyber threats and ensures data security through robust M2M connectivity	−	−	−	−	−
	[45]	2021	Overview of the integrity of industrial IoT systems, classifying various attacks and security solutions: IoT/IIoT security solutions include communication protocols, networks, cryptography, and IDS	−	−	+	−	+
	[123]	2024	Data filtering, encryption, and decentralized processing strengthen IIoT systems against cyber threats	−	−	−	−	+
	[134]	2024	A real-time security system using digital twin technology and interactive ensemble ML to enhance attack detection in IIoT environments and tackle data-related issues	+	−	+	−	−
	[156]	2024	IoT-Defender, with MGA for feature selection and LSTM for cyberattack detection in IoT networks, aims to enhance IDS performance by optimizing relevant feature selection	+	−	+	−	−
	[171]	2024	A secure M2M authentication protocol in IIoT utilizing ECC-based cryptography to enhance security	−	−	−		+
	[172]	2024	Blockchain-based IIoT architectures enhance security and privacy while developing a reputation-based behavioral punishment mechanism to improve security effectiveness	−	+	−	−	−
	[173]	2023	Network-level security for protecting production through virtualization, designed for legacy environments, aims to converge IT and OT domains to enhance scalability and security in manufacturing networks	−	−	−	+	−
IIoT–Edge Computing	[5]	2022	A framework designed for the secure transmission, storage, and computation of IIoT tasks that combines edge computing with IIoT platforms. It employs simplified encryption and a modified ElGamal encryption method, along with digital signatures, to improve overall performance.	−	−	−	+	−
	[122]	2024	FL platform that optimizes industrial data processing with minimal latency and security, addressing efficient data processing for manufacturers in smart production environments with edge technology support	+	−	−	−	−
	[153]	2024	AI improves diagnostic and predictive methods for industrial machines, addressing privacy issues, high latency, and low availability through edge-level computations	+	−	−	−	−
	[154]	2024	Blockchain-based FL enhances collaborative intrusion detection in IIoT environments by ensuring data privacy and reducing vulnerability to MITM attacks through a secure parameter verification scheme. The architecture improves intrusion detection accuracy	+	−	−	−	−
	[174]	2023	Security threats in the edge computing–IIoT environment include access control, encrypted communication, and authentication measures	−	−	−	−	+
CPs IIoT–Edge Computing	Our paper	2024	Common attacks to IIoT–edge computing highlight various cyberattacks on CPS and their industrial impact. They underscore the importance of integrating IIoT–edge computing for protection against CPS IIoT cyberattacks. A taxonomy of main security methods for CPS IIoT–edge computing has been developed, comparing our approach with other sources in this research area	+	+	+	+	+

## Data Availability

The original contributions presented in this study are included in the article. Further inquiries can be directed to the corresponding author.
